# Community violence intervention: measuring risk & protective factors for gun use among program participants

**DOI:** 10.1186/s40352-025-00393-x

**Published:** 2026-01-26

**Authors:** Jason Corburn, Alice Bruno, Juan Cabrera, Sean Darling-Hammond, Mahasin Mujahid

**Affiliations:** University of California, Berkeley, Berkeley, United States

**Keywords:** Firearm, Gun, Community violence, Risk factors

## Abstract

Community Violence Intervention (CVI) involves recruiting likely gun violence offenders using street outreach and offering mentorship, social services and other supports to discourage future firearm violence. Grounded in public health, CVI interventions identify an individual’s risk factors and work to enhance protective and buffering factors that can help prevent a client from offending. This paper reviews evaluations of CVI interventions to understand how participants’ risk factors are defined and measured. We used keywords to identify 38 published evaluations of 32 different CVI interventions that recruited community members and used street-outreach as the primary mode of engagement. We then used the PRISMA scoping review methodology to identify categories of risk and protective factors to screen each published evaluation for whether and how they measured participants’ risk, protective and buffering factors at program onset. We found that 56% (18/32) of evaluations included some demographic information about participants and 44% (14/32) provided additional details about their educational or employment status. The criminal justice history was reported in 59% (19/32) and gang or group affiliation in 47% (15/32) of the evaluations we reviewed. The CVI participants’ history related to gun violence was documented in 53% (17/32). Far fewer evaluations documented potential assets, buffering or protective factors of CVI participants: only 16% (5/32) reported on whether any clients were receiving social services at intake and only 6% (2/32) documented any clients’ strengths or protective factors. We also found that no two programs captured the same information about gang, criminal justice, gun violence or social risk factors. Preliminary findings suggest that many CVI programs may not be capturing whether clients face known risk factors for future gun use, and that these programs rarely capture potential protective and buffering factors that might inform program service delivery and potentially enhance the likelihood that interventions will help prevent future gun use. This study aims to support the emerging field of CVI as it works to build evidence that these interventions successfully address the specific needs of urban young people and prevent them from engaging in firearm offending.

## Introduction

Urban gun violence continues to be an epidemic disproportionately affecting racially marginalized communities in the United States. For children and adolescents, firearm-related injuries have been the leading cause of death since 2020, with African Americans in this age group experiencing a gun death rate 18 times higher of similar white children (Villareal et al., 2024). The disparities persist among young adults, and in 2022 African American men under 35 years old accounted for 34% of all gun homicides (Marineau et al., 2024). The focus of this review, community firearm violence, occurs in public settings between individuals and small groups that may or may not know one another, is often unplanned and impulsive, and both perpetrators and victims in the US are frequently, but not exclusively, young Black men from disadvantaged backgrounds and communities Carter et al., (2020). The US Surgeon General issued an Advisory on Firearm Violence in 2024, noting that the inequitable distribution of community firearm violence – and, by extension, its myriad social and health repercussions – is related to decades of structural racism and disinvestment (Office of the Surgeon General, 2024).

Community Violence Intervention (CVI) has gained increasing recognition as a public health and evidenced-based approach with the potential to disrupt gun violence and violent behaviors by directly engaging with individuals involved in cyclical and retaliatory violence (DOJ, 2025).

One key tenet of CVI is that individual behavioral changes can prevent future gun use, and programs deliver a set of services and supports to mitigate risks and build on protective factors to help induce these changes. In this paper, we suggest that knowing about a CVI participant’s baseline characteristics—when they enter a program—can help the field assess how risks are effectively addressed, whether strengths and assets are leveraged, and what supports and services might be helping promote peaceful behavioral changes. Thus, we conduct a scoping review of published CVI evaluations focused on street-outreach engagement to investigate what information about participants’ risk and protective factors is reported. We ask whether risk factors known to be associated with gun use, such as Adverse Childhood Experiences and prior gun use and exposure, are routinely reported and if the field of CVI might begin to develop a set of shared measures of risk and protective factors to better understand participant needs, identify opportunities to more effectively intervene, and determine which violence prevention measures can sustain peace. Our aim is to inform the emerging field of CVI and support the notion that effective interventions, particularly those employing a public health approach, ought to be informed by pre-existing risk and protective factors.

### Defining community violence intervention

Guidance issued by the White House in 2021 defined CVI as including “focused deterrence, street outreach, and hospital-based violence intervention models, complete with wraparound services such as behavioral therapy, trauma recovery, job training, education, housing and relocation services, and financial assistance” (Department of the Treasury, 2021). While there is no singular definition of CVI, a 2024 ‘CVI Action Plan’ co-authored and endorsed by 135 organizations and individuals in the field, defined it as a community-centered approach to reducing violence by: (a) engaging very high-risk individuals and groups to disrupt cycles of violence and retaliation (b) using street outreach workers to establish relationships between individuals and community assets to deliver services that save lives, address trauma and provide opportunity; and, (c) employing relational strategies including, but not limited to, violence interruption, hospital-based violence intervention, life coaching, peace fellowships, violence-focused cognitive behavioral therapy, and others (CVI Action Plan, 2024).

This paper focuses on street outreach-based CVI strategies, which seek out individuals that are actively engaged in, or suspected of being at high risk for, gun violence. These same CVI programs intervene by first building a trusting relationship between participants and their assigned street outreach worker, most of whom are defined as ‘credible messengers’ since they are formerly incarcerated community members who have returned from prison with mentorship, social emotional intelligence, conflict mediation and other counseling and social work-type skills (Szkola & Blount-Hill, 2024). The outreach workers either deliver trauma-informed services themselves or make referrals to third-party providers, often making decisions about needed service types and intensity based on individual risk, community context, immediate group dynamics, and participant readiness (Hureau & Papachristos, 2024).

CVI is explicit that the approach to preventing gun violence is grounded in public health (CVI Action Plan, 2024). A public health approach to reducing urban gun violence in the US includes efforts that combine identifying and measuring factors that raise or reduce risk for firearm use, instituting policies and practices that eliminate risk and increase protective factors, helping those already harmed to heal and avoid retaliatory actions, reducing access to instruments of harm, and evaluating interventions for their effectiveness (Byrdsong et al., 2016; Decker et al., 2018). The 2002 World Health Organization’s World Report on Violence and Health suggested that the first step in a public health approach to violence prevention is to identify “biological and personal factors that influence how individuals behave and increase their likelihood of becoming a victim or perpetrator of violence: demographic characteristics (age, education, income), personality disorders, substance abuse, and a history of experiencing, witnessing, or engaging in violent behaviour” (Krug et al., 2002, p.1085). Webster (2022, p.39) notes that “public health models for violence prevention also seek to support those at greatest risk of violence by addressing factors that elevate the risk of violence.” Buggs et al., (2022a, p.2) suggested that there is a “need for tailored interventions aimed at identifying and engaging youth at greatest risk of firearm violence exposure in affordable, accessible, and culturally responsive mental health support services in their schools and communities.”

CVI evaluations have returned mixed results concerning whether changes in participants’ behaviors contribute to reductions in gun violence (e.g., Braga et al., 2019c; Butts et al., 2015; Chwalisz, 2023; Cure Violence, 2021; McGarrell et al., 2009; Ross et al., 2023; Skogan et al., 2008; Webster et al., 2012). A scan of 14 community-based youth gun violence prevention programs by the *Urban Institute* found that all used individual case management, most catered interventions to the needs and interests of clients, and all regretted not having data available to ‘prove’ their interventions were effective in changing behaviors and reducing violence (Matei et al., 2022, p. 36). Hureau et al., (2023, p.771) noted that gun-violence prevention street outreach programs have failed to document “behavioral change among the people or groups theorized to be driving neighborhood and population rates of violence…” and that “researchers have missed a crucial opportunity to develop knowledge on how such interventions influence the behavior of those most proximate to them.” Bhatt et al. (2024) noted in their evaluation of READI Chicago that the first challenge in preventing future shootings is identifying the complex mix of trauma and social disconnection that tends to predict which individuals are most at risk of engaging in gun violence.

### Risk and protective factors for engaging in community gun violence

Researchers have demonstrated that there is no single exposure, family dynamic, school experience, peer influence, or sociocultural inequity that accounts for why someone will use a gun (Altikriti & Connolly, 2024; Kinscherff et al., 2013; Wintemute, 2015). Rather, engaging in gun violence is often associated with a confluence of individual, family, school, peer, community, and institutional factors that interact over time – potentially from in-utero – to adversely impact decision-making (Kim, 2019; Sanchez et al., 2020). Exposure to multiple forms of violence (i.e., poly-victimization) is associated with a graded dose–response relationship with violent behaviors (Davis et al., 2018). Multiple studies have demonstrated that concentrated, multi-generational structural disadvantages – including exposure to gun violence, poverty, residential segregation, and limited prospects for upward mobility – remain key drivers behind the disproportionate burdens of firearm homicides and injury shootings experienced by young Black men in the US (Buggs et al., 2022a; Houghton et al., 2021; Johnson et al., 2021). Importantly, individual-level risk and protective factors tend to interact in complex ways within one’s peer and neighborhood networks, suggesting that they are important for identifying *which* young people from disadvantaged areas are more likely to use guns absent any ameliorative interventions (Schmidt et al., 2019).

A study by Pardini et al. (2021) reported that male youth most likely to commit gun offenses were those who routinely carried guns, associated with gun-carrying peers, belonged to gangs or groups that rewarded criminal activity, and engaged in drug dealing and heavy alcohol use. Carter et al. (2020) found that—among young Black males who had sought treatment in an urban emergency department—having substance use issues, having positive attitudes toward firearm use, having peers who also carried weapons, and living in areas with high levels of community violence were the key risk factors. Papachristos et al. (2015) found that when an individual is shot, the risk of being shot goes up exponentially among the victim’s social network with whom they have engaged in criminal activity, especially among those closest to the victim. Others have shown that being a member of a street group that has known rivals can lead to ongoing victimization and may result in retaliation (Park et al., 2021). However, some studies suggest that just being affiliated with a ‘gang’ or ‘group’ does not necessarily predict criminal or violent behaviors (Barnes et al., 2010).

Prior arrest for carrying and using a gun, delinquent peer affiliations, community violence exposure, and attitudes toward retaliation have also been shown to increase the likelihood of a group-affiliated individual from using a gun to resolve a conflict in the future (Carter et al., 2020; Gonzales & McNiel, 2020; Teplin et al., 2021). Combinations of experiencing homelessness, childhood trauma, drug use and prior victimization have been associated with male gun violence involvement (Goldstick et al., 2019; Hsu et al., 2021; Pardini et al., 2021). Victimization contributing to future lethal violence can range from experiences of being robbed, physically and/or sexually assaulted, bullied, or discriminated against (Lord et al., 2002). Some, but not all, assault victims and those who are fearful of being victimized carry guns for self-protection and may subsequently use them. For example, a systematic review noted that when young people do not have access to positive resources (i.e., health, nutritional, educational, recreational, or financial), have poor social relationships with others, or feel unsafe in their environment, the likelihood of involvement in behaviors like gun violence increases (Sanchez et al., 2020).

Another well-documented risk factor for gun violence is prior Adverse Childhood Experiences, or ACEs (Gilbert et al., 2023; O'Connor et al., 2021; Wamser-Nanney et al., 2019). ACEs can contribute to posttraumatic stress, emotional numbing, adversely impact impulse control, a constant survival mentality and induce anxiety that can make it difficult to interpret whether situations and people are safe or a threat, all of which can contribute to toxic stress and decisions to use a gun to address conflicts (Gaylord-Harden et al., 2022). Rajan et al. (2019) found that simultaneously experiencing multiple ACEs (including child maltreatment, household dysfunction and domestic violence) and high levels of community gun violence increased the likelihood of future violent behavior. Jones et al. (2023) found that the more ACEs an adolescent was exposed to, the more likely they were to have carried a handgun in the past 12 months.Others have found that the influence of ACEs on future gun use can differ by age (Baglivio et al., 2020). The research on the links between ACEs and preventing gun use suggests the importance of understanding the presence and influence of unaddressed trauma and related depression, threat sensitivity, and emotional distress (Altikriti et al., 2025).

Notably, ACEs do not include measures of exposure to gun violence, such as a prior gun injury, frequently hearing gunshots, carrying a gun, or witnessing a gun homicide, all of which are also documented risk factors for future gun use (Beardslee et al., 2018; Mitchell et al., 2021). Youth exposed to neighborhood gun violence were twice as likely to carry a weapon when compared to those not exposed to it in their communities (Baiden et al., 2024). Higher prevalence of guns in a neighborhood, and their ease of accessibility, also predicted future gun use among adolescents (Gonzales & McNiel, 2020). Still, few studies consistently measure community gun violence exposure, capture the differential impacts of exposure to gun violence among different age groups, or detail how indirect gun violence exposure (i.e., hearing gun shots, witnessing gunfire, knowing someone who was shot, or being aware of gun violence in one’s community) contributes to trauma, poor mental health, and may contribute to future decisions to use a firearm (Bancalari et al., 2022; Shulman et al., 2021).

ACEs also fail to capture structural violence, or the ways in which inequitable power imbalances become normalized and reinforced by institutions, often appearing routine, and ultimately benefiting some groups while oppressing others by limiting access to basic needs (Farmer, 2004; Galtung, 1969). Structural violence can influence gun violence through place-based risks such racial residential segregation, chronic poverty, environmental hazards, under-resourced schools, limited safe and green community spaces, and dilapidated and unaffordable housing (Buggs et al., 2023). Community-level factors, such as ease of accessing alcohol outlets, neighborhood disorganization, low collective efficacy, racial segregation and impoverishment, can compound the effects of individual- and peer-level risks for gun use (Dong et al., 2017; Gard et al., 2022; Tracy et al., 2019). Neighborhood blight and physical disorder, including disproportionate numbers of dilapidated buildings and vacant lots, have been shown to predict firearm violence (Branas et al., 2018). Living in a neighborhood with chronic social and economic disadvantage can increase the likelihood of being involved in community gun violence (Beardslee et al., 2021). Individual basic need insecurities (i.e., food, clothing and shelter) and area-based concentrated disadvantage together can increase the risk of future violence exposure and involvement (Miller et al., 2021).

A public health approach to preventing gun violence would also capture and seek to understand the presence or absence of protective (risk avoidance) and buffering (risk mitigating) factors for individuals at the center of gun hostilities (Bernat et al., 2012). Protective factors are an aspect of strengths-based prevention approaches and seek to leverage and reinforce positive attributes and resources that might promote peaceful conflict resolution and human development (Fortune, 2018; Ttofi et al., 2016). Some known and suspected factors that protect against engaging in violence include the presence of healthy, available adults (Ashton et al., 2021), nurturing relationships (Jaffee et al., 2013), having supports for aspirational goals (Knight et al., 2017), and repeated exposure to positive community experiences or environments (PCEs) (Bethell et al., 2019; Yu et al., 2022). However, there is limited evidence on whether single or combinations of protective/buffering factors, occurring at different stages of the developmental period, might help prevent gun use (Mattson et al., 2020).

Finally, primary care and emergency department practitioners have developed screening tools to identify whether a gunshot victim is likely to use a gun to retaliate or become a repeat victim (Goyal et al., 2023). For example, the *SaFETy* score tool uses four questions – violence victimization, community exposure to gun violence, peer influences and fighting – to predict future firearm violence risk (Goldstick et al., 2017). Another gun violence risk assessment instrument, the Violent Reinjury Risk Assessment Instrument (VRRAI), uses 29 questions to assess the presence of risk indicators, behavioral factors, and environmental factors that may signal an elevated risk of reinjury for patients (Kramer et al., 2017). These and related assessment tools emphasize that interventions should be informed by and tailored to the individual’s risk context, abilities, and strengths (Armstrong et al., 2023). Given these and related gun violence risk assessment tools, we would expect that community violence intervention and prevention strategies would borrow from available risk-assessment tools and similarly assess and document the individual, peer, community and environmental risk and protective factors that their clients may be experiencing prior to treatment (Bonfine et al., 2021; Borum et al., 2021; Stockdale, 2019).

## Methods

We conducted a ‘scoping review’ (Levac et al., 2010) following the PRISMA-ScR guidance to better understand the kinds of participant information available within published CVI evaluations (Tricco et al., 2018). A scoping review is particularly well-suited for this inquiry as it aims to develop an “overview of the evidence rather than a quantitative or qualitative synthesis of data” and “identify, present and describe relevant characteristics of included sources of evidence rather than seeking to combine statistical or qualitative data from different sources to develop synthesized result” (Peters et al., 2021, p.2). This approach allows for the examination of a diverse and evolving body of literature, making it ideal for mapping how “at-risk” individuals are defined, characterized, and operationalized in CVI evaluations.

### Eligibility criteria, search, and selection of sources

Given the multifaceted nature of these programs, CVI evaluations are published in a broad range of scholarly and non-scholarly sources, spanning from peer-reviewed journals to organization websites to government and funding agency reports. Thus, we developed a comprehensive search strategy that would allow us to survey academic sources, as well as reports published elsewhere. We began by conducting searches in 11 academic databases to identify peer-reviewed evaluations and complemented our results by surveying grey literature sources such as government and CVI organizations’ websites. The search was conducted between April and July 2025.

We developed a keyword search strategy through preliminary testing in Web of Science and refined search terms based on the results. The initial choice of keywords was informed by the definitions used by CVI practitioners (i.e., CVI Action Plan, 2024), combined with Boolean operators. Search terms included words related to: (1) CVI programs, such as “Community Violence Intervention”, “Group Violence intervention”, “Focused Deterrence”,”Cure Violence”, and “Operation Ceasefire”; (2) unique CVI program identifiers, such as “street outreach” and “credible messenger”; and (3) evaluation language, such as “program effectiveness”, “intervention evaluation”, “effectiveness”, and “impact.” Once we identified a final search strategy, we extended it to other databases and adapted it for website search functions. The databases included: Criminal Justice Abstracts, PubMed, JSTOR, Social Sciences Full Text, Sociological Abstracts, Criminal Justice Abstracts, National Criminal Justice Reference Service Abstracts, Proquest Criminal Justice, SciELO, and Google Scholar.

Since we were interested in reviewing street outreach-focused CVI programs, we excluded terms associated with ‘hospital-based violence intervention programs (HVIP),’ although they are often considered part of the CVI ecosystem (Nofi et al., 2023). This choice was based on the fundamental difference in data access: HVIPs tend to recruit participants from clinical settings, where they have access to built-in healthcare data systems mandated to collect participant information, whereas the focus of this review is on community-based programs without a priori access to participant data. Moreover, we limited our search to publications in English and Spanish from 2010 onwards to focus on contemporary CVI programs and evaluation practices. Lastly, we excluded the search terms related to ‘Advance Peace,’ the ‘peacemaker fellowship,’ and ‘Richmond, Office of Neighborhood Safety,’ due to the authors’ ongoing involvement in the data collection processes and evaluation of these CVI programs. Upon reviewing the results, we further refined our search by excluding papers in the domains of international conflict, intimate partner violence, or domestic violence. The complete search strategy is provided in Appendix A.

**Fig. 1:**
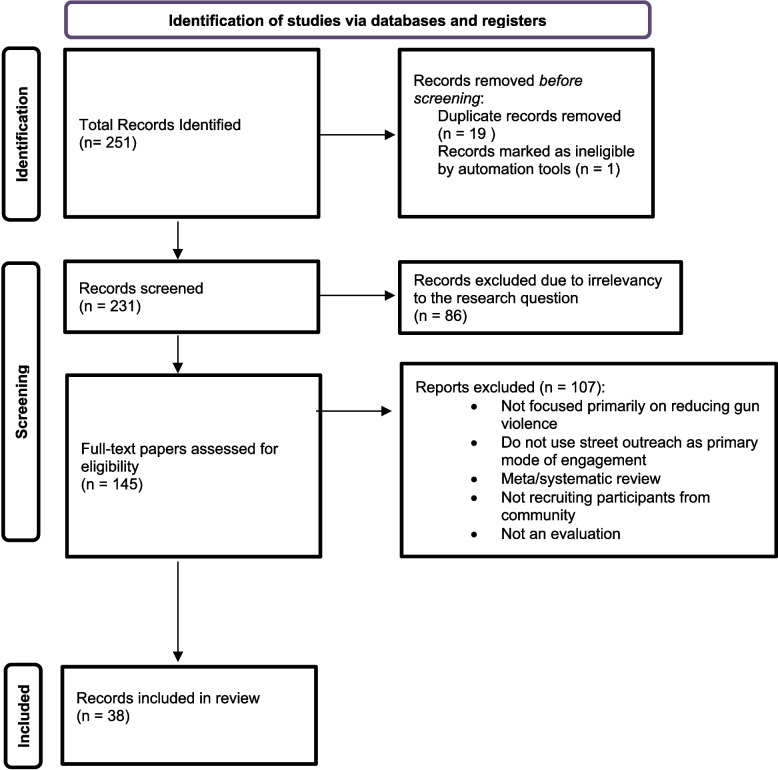
PRISMA Diagram of CVI Evaluation Selection Criteria, 2010–24

Search results were complemented with hand-searching references from identified articles and reports. We included all evaluations regardless of study design or quality of evidence. The final list of sources was first screened by title to identify records irrelevant to the research question. Full text and supplemental files were analyzed for the remaining sources to assess eligibility, and a total of 38 records met all eligibility criteria for inclusion in the review (Table 1). Figure 1 shows a PRISMA diagram of the process (Page et al., 2021).

**Table 1:** CVI evaluations reviewed for participant data

**Location**	**Programs Implemented (*n* = 32)**	**Report/Paper Title (*n* = 38)**	**Author (Year)**
Baltimore, USA	Safe Streets	Evaluation of Baltimore's Safe Streets Program: Effects on Attitudes, Participants' Experiences, and Gun Violence	Webster et al. (2012)
		Effects of Baltimore’s Safe Streets Program on Gun Violence: A Replication of Chicago’s CeaseFire Program	Webster et al. (2013)
		Estimating the Effects of Law Enforcement and Public Health Interventions Intended to Reduce Gun Violence in Baltimore	Webster et al. (2018)
		Using Synthetic Control Methodology to Estimate the Effects of a Cure Violence Intervention in Baltimore, Maryland	Buggs et al. (2022b)
		Estimating the Effects of Safe Streets Baltimore on Gun Violence 2007–2022	Webster et al. (2023)
	Ceasefire 365	Baltimore Ceasefire 365: Estimated Impact of a Recurring Community-led Ceasefire on Gun Violence	Phalen et al. (2020)
	ROCA	Roca Baltimore End of Year Report, Fiscal Year 2023	Roca (2023)
Boston, USA	Operation Ceasefire	Deterring Gang-Involved Gun Violence: Measuring the Impact of Boston’s Operation Ceasefire on Street Gang Behavior	Braga et al. (2014)
	ROCA	Final Report: Phase II Evaluation of Roca's CBT Curriculum	Abt Associates (2021)
Cali, Colombia	Abriendo Caminos (Cure Violence)	Informe Final de la Evaluación de Impacto del Programa Abriendo Caminos de la Fundación Alvaralice	Leon et al. (2020)
Cambridge, USA	Focused Deterrence	Focused Deterrence: Effective Crime Reduction Strategy for Chronic Offenders?	Schnobrich-Davis et al. (2021)
Chicago, USA	Create Real Economic Destiny (CRED)	Evaluating the Impact of a Street Outreach Intervention on Participant Involvement in Gun Violence	Ross et al. (2023)
	Rapid Employment and Development Initiative (READI)	Predicting and Preventing Gun Violence: An Experimental Evaluation of READI Chicago	Bhatt et al. (2024)
	Communities Partnering 4 Peace (CP4P)	Communities Partnering 4 Peace 2018—2023, Five Year Research & Evaluation Report	Center for Neighborhood Engaged Research & Science (CORNERS) (2023)
Denver, USA	Gang Reduction Initiative of Denver (GRID)	Multidisciplinary Teams, Street Outreach, and Gang Intervention: Mixed Methods Findings from a Randomized Controlled Trial in Denver	Pyrooz et al. (2023)
Indianapolis, USA	Coalition to Advance Public Safety (CAPS)	Indianapolis Violence Reduction: Assessment & Recommendations by the National Institute for Criminal Justice Reform	National Institute for Criminal Justice Reform (2020) & (2025)
		Indy Coalition to Advance Public Safety (CAPS) Impact Report	
Illinois, USA	Northern Illinois Project Safe Neighborhoods (PSN)	Improving Programming in Juvenile Detention: The Impact of Project Safe Neighborhoods Youth Outreach Forums	Davis et al. (2025)
Kansas City, USA	No Violence Alliance	Collaborating to Reduce Violence: The Impact of Focused Deterrence in Kansas City	Fox and Novak (2018)
Los Angeles, USA	Gang Reduction and Youth Development (GRYD)	Evaluation of the Los Angeles Gang Reduction and Youth Development Program Year 4 Evaluation Report	Cahill et al. (2015)
		The Impact of the City of Los Angeles Mayor’s Office of Gang Reduction and Youth Development (GRYD) Comprehensive Strategy on Crime in the City of Los Angeles	Brantingham et al. (2021)
New Haven, USA	Project Longevity	Evaluating the Effect of Project Longevity on Group-Involved Shootings and Homicides in New Haven, CT	Sierra-Arevalo et al. (2015)
New Orleans, USA	Focused Deterrence	Most Challenging of Contexts: Assessing the Impact of Focused Deterrence on Serious Violence in New Orleans	Corsaro and Engel (2015)
New York, USA	Save Our Streets (Cure Violence in Crown Heights, NYC)	Testing a Public Health Approach to Gun Violence: An evaluation of Crown Heights Save Our Streets, a replication of the Cure Violence Model	Picard-Fritsche & Cerniglia (2013)
	Save Our Streets (Cure Violence in South Bronx and East New York, NYC)	Denormalizing Violence: The Effects of Cure Violence in the South Bronx and East New York, Brooklyn	Delgado et al. (2017)
Newark, USA	Newark Community Street Team (NCST)	The Newark Community Street Team Narrative Evaluation	Leap et al. (2020)
Oakland, USA	Ceasefire	Oakland Ceasefire Evaluation Final Report to the City of Oakland	Braga et al. (2019a)
	Focused Deterrence	Street Gangs, Gun Violence, and Focused Deterrence: Comparing Place-based and Group-based Evaluation Methods to Estimate Direct and Spillover Deterrent Effects	Braga et al. (2019b)
	Oakland Unite	Oakland Unite 2016–2020 Comprehensive Evaluation: Implementation and Impacts of Youth and Adult Life Coaching	Gonzalez et al. (2021)
Pittsburgh, USA	One Vision One Life	Community-Driven Violence Reduction Programs: Examining Pittsburgh’s One Vision One Life	Wilson and Chermak (2011)
Philadelphia, USA	Ceasefire	Philadelphia CeaseFire Findings from the Impact Evaluation	Roman et al. (2017)
	Group Violence Intervention (GVI)	Evaluation of the Current Group Violence Interventions (GVI) Implementation in Philadelphia	Moyer (2023)
	Community Expansion Grant (CEG) Antiviolence Initiative	Evaluation of the City of Philadelphia Community Expansion Grant (CEG) Antiviolence Initiative	City of Philadelphia (2023)
Phoenix, USA	TRUCE	Evaluation of the Phoenix TRUCE Project: A replication of Chicago Ceasefire	Fox et al. (2015)
Stockton, USA	Focused Deterrence	Focused Deterrence, Strategic Management, and Effective Gun Violence Prevention	Braga et al (2024)
Tampa, USA	Project Safe Neighborhoods	Evaluating the impact of Project Safe Neighborhoods (PSN) Initiative on Violence and Gun Crime in Tampa: Does it Work and Does it Last	Fox et al (2022)
Trinidad and Tobago, USA	Cure Violence	Evaluating Cure Violence in Trinidad and Tobago	Maguire et al. (2018) & Adams and Maguire (2023)
Washington, D.C, USA	Cure the Streets	Beating the Gun—One Conversation at a Time? Evaluating the Impact of DC’s “Cure the Streets” Public Health Intervention Against Gun Violence	Chwalisz (2023)

### Charting the data

Following the scoping review guidelines, we designed a protocol to extract and analyze data relevant to the research question before conducting our review. This process, known as “data charting,” is iterative and entails continuous updating of the information to chart based on learnings from the review (Mak & Thomas, 2022). Two co-authors, (JC & AB) performed the data charting. A first set of data extraction fields were defined a priori, informed by a review of the literature on risk and protective factors associated with firearm activity, as presented earlier in the paper. As noted above, some well documented risk factors for using guns in community violence include being a young male, African American, participating in criminal activity, prior arrests, low levels of education, being disconnected from social services, living in poverty, prior drug dealing, gang/group membership, gun carrying, witnessing gun violence and prior victimization. Consistent with the iterative methodology of scoping reviews, we revised the original charting fields after independently reviewing all sources to capture the breadth of evidence and information presented accurately. The final extraction fields are shown in Table 2. Using the final extraction fields, we conducted an independent, in-depth review of the sources. We then discussed the analysis results, cross-checked our findings, and resolved inconsistencies.

**Table 2:** Screening criteria used to characterize participants’ risks in CVI evaluations

Risk/Protective category	Variable/Characteristic
Demographics	• Race/Ethnicity; age; gender • Employment & educational status • Residential location
Gang or Group Affiliation	• Active/known or suspected gang membership •Information about rival/warring groups
Criminal Justice System	• Prior Incarceration • Prior arrests but not incarcerated • On parole/probation • Juvenile detention/incarceration
Gun Violence Exposure	• Witness to a gun homicide • Frequent gun violence exposure • Gunshot injury • Peer gun carrying
Social risks/ victimization	• Household dysfunction • School suspension/discipline • Substance abuse/drug addiction • History of victimization, not gun (i.e., physical assault, abuse) • Housing/homelessness status • Experience in foster care • Food security status • Area-based deprivation/disinvestment
Receiving Services	• Enrolled in/receiving public assistance • Receiving or ever enrolled in counseling • Medicaid/health care access
Assets/Protective Factors	• Any talents/skills, including job/employment related • Current employment status • Is a parent • Engaged in pro-social activities/Strong executive function

## Results

Our review reveals heterogeneous reporting of program participant risk and protective factors at program inception. We found that 56% (18/32) of the evaluations in our sample reported some, although not consistent, information on participants’ demographics, while 44% (14/32) reported on participants’ employment and/or educational status. We found that 59%** (**19/32) reported information on the prior criminal justice involvement, but only 47% (15/32) reported on whether participants were gang or group affiliated. We found that 53% (17/32) reported information on whether or not a participant had any prior gun violence exposures, and only 25% (8/32) reported on prior adverse social/environmental exposures. Finally, 16% (5/32) reported whether any clients were receiving social services at intake, and 6% (2/32) documented any strengths or assets of participants at enrollment (Table 3). Denver’s GRID and Los Angeles GRYD were the only two evaluations that met all our criteria for reporting information about clients’ risks and protective factors.

**Table 3:** CVI Evaluations reporting participant characteristics at intake

Criminal Justice System	Demographics	Gun Violence Exposure (Victimization)	Gang or Group Affiliation	Education & Employment	Social & Environmental Risks	Receiving Services	Assets & Protective Factors
19 (59%)	18 (56%)	17 (53%)	15 (47%)	14 (44%)	8 (25%)	5 (16%)	2 (6%)
· Baltimore, ROCA · Baltimore, Safe Streets · Boston, ROCA · Chicago, CP4P · Chicago, CRED · Chicago, READI · Denver, GRID · Illinois, PSN · LA, GRYD · NYC, Save Our Streets Crown Heights · NYC, Save Our Streets, South Bronx & East NY · Oakland Unite · Oakland, Ceasefire · Pheonix, TRUCE · Philadelphia, CEG · Philadelphia, GVI · Stockton, Focused Deterrence · Tampa, PSN · Trinidad, Cure Violence	· Baltimore, ROCA · Baltimore, Safe Streets · Boston, ROCA · Chicago, CP4P · Chicago, CRED · Chicago, READI · Denver, GRID · Illinois, PSN · LA, GRYD · NYC, Save Our Streets Crown Heights · NYC, Save Our Streets, South Bronx & East NY · Oakland Unite · Oakland, Ceasefire · Pheonix, TRUCE · Philadelphia, CEG · Philadelphia, GVI · Stockton, Focused Deterrence · Trinidad, Cure Violence	· Baltimore, ROCA · Baltimore, Safe Streets · Chicago, CP4P · Chicago, CRED · Chicago, READI · Denver, GRID · LA, GRYD · NYC, Save Our Streets Crown Heights · NYC, Save Our Streets, South Bronx & East NY · Oakland Unite · Oakland, Ceasefire · Pheonix, TRUCE · Philadelphia, CEG · Philadelphia, GVI · Stockton, Focused Deterrence · Tampa, PSN · Trinidad, Cure Violence	· Baltimore, ROCA · Boston, Operation Ceasefire · Boston, ROCA · Chicago, CRED · Denver, GRID · KC, No Violence Alliance · LA, GRYD · New Haven, Project Longevity · New Orleans, Focused Deterrence · NYC, Save Our Streets Crown Heights · Oakland, Ceasefire · Pheonix, TRUCE · Stockton, Focused · Deterrence · Tampa, PSN · Trinidad, Cure Violence	· Baltimore, ROCA · Baltimore, Safe Streets · Boston, ROCA · Chicago, CP4P · Chicago, CRED · Chicago, READI · Denver, GRID · Illinois, PSN · LA, GRYD · NYC, Save Our Streets Crown Heights · NYC, Save Our Streets, South Bronx & East NY · Oakland Unite · Pheonix, TRUCE · Philadelphia, CEG	· Baltimore, ROCA · Baltimore, Safe Streets · Boston, ROCA · Chicago, CRED · Denver, GRID · LA, GRYD · Pheonix, TRUCE · Philadelphia, CEG	· Baltimore, ROCA · Boston, ROCA · Denver, GRID · LA, GRYD · Oakland Unite	· Denver, GRID · LA, GRYD

### Demographics/education/employment

Demographic information about participants included their age, ethnicity, and gender, while educational status/attainment included such information as whether or not the participant was currently in school, their last grade attended, if they completed high school, a GED, or a trade school, and their highest level of education. LA’s GRYD was the only program to document whether or not a participant had a documented learning disability and information about their academic performance, while Oakland’s Unite and LA’s GRYD were the only programs in our sample to report if a participant had been suspended from school and/or was involved in a violent incident at school.

The employment measures were similarly nuanced, but only 7/14 in this category reported whether or not the participant was employed at the time of enrollment. One program, Communities Partnering for Peace in Chicago, recorded whether the employment was full or part-time, and only Phoenix’s TRUCE reported whether or not the participant had ever received a referral for employment. We found no programs that assessed participants’ job skills.

We found some program evaluations provided detailed descriptions of demographic information, while others offered only generalities. For example, Denver’s GRID characterized participants as “a young adult male who is gang-involved…The average age of a referral is age 20 years, though the bulk of sample (71 percent) was juvenile. GRID’s clientele is overwhelmingly Hispanic (49 percent) or Black (34 percent), and male (99 percent)” (Pyrooz et al., 2023, p. 21). Chicago’s CRED described participants as “all […] Black, the average participant age at intake is roughly 24 years old, and the average level of completed education was 11 years of school.” Washington, DC’s, Cure the Streets program specified that age and gender were eligibility criteria for participants, stating that they only targeted “high risk male youth, aged 18 to 24,” but failed to provide any additional demographic detail of the enrolled participants. We found only Oakland’s Unite, Denver’s GRID and Chicago’s CRED published data that included quantitative detail on participants’ demographics.

### Criminal justice system/gang group affiliation

We found that 59% of the evaluations we reviewed included some descriptive information about participants’ criminal legal history. The category ‘criminal justice system’ included information including any criminal background, such as prior arrest, convictions, incarceration, and/or youth authority detention. Fifteen evaluations reported whether participants had ever been arrested, and ten of these programs asked whether the arrest (but not always conviction) was firearm and/or violent crime related. For example, Boston’s ROCA, Cure Violence Trinidad, CP4P, CRED, READI, GVI Philadelphia, Denver’s GRID, and Oakland’s Unite, captured whether participants had a prior weapons-related (i.e., shooting or homicide) arrest and/or conviction. Seven evaluations captured information about prior incarceration, but only CRED Chicago and Oakland’s Unite included information on whether participants had experienced detention as a juvenile.

Whether or not participants were gang or group-affiliated was documented in thirteen of the evaluations. Some programs, such as LA GRYD, Oakland Ceasefire, NY’s Save our Streets, and Denver’s GRID, define themselves as gang-reduction programs and thus only recruit from these populations. However, we found inconsistent reporting even across gang-focused intervention programs. For example, Denver’s GRID and LA’s GRYD included participant data at intake about not just an individual’s affiliation but also on what they called ‘intergenerational gang membership’ among family and peers. The evaluation for Oakland Unite did not report the specific number of participants who were gang members, but the descriptive text states that the program utilizes “data-driven risk factors” to identify participants, such as group/gang involvement. The Tampa PSN program developed what they called the Violent Impact Player List (VIP List) to characterize and identify firearm offending risks. The VIP List included assigning points and a total ‘risk score’ based on whether a community member had one or more of the following: (a) prior firearm offense/arrest (b) violent criminal history (c) gang member/affiliation within past 5 years (d) probation or release from prison within past 3 years (e) was a suspect in a shooting, an associate of a suspect in shooting, or was a victim in a shooting themselves, and (f) felony nonviolent arrests within past 2 years (Fox et al., 2022, p.549).

The supplemental material provided in the evaluation of Chicago’s CRED noted that most CVI evaluations rely on police department-defined gang/group affiliations (i.e., document in an administrative data set), not local knowledge of outreach workers, and this is controversial and potentially inaccurate. The CRED evaluation also notes: “CPD[Chicago Police Department]-defined gang members have on average, more arrests, victimizations, and co-arrestees in the three years prior to their imputed start dates compared to non-gang members. This means that, even though CPD-defined gang affiliation is indeed flawed, it likely captures some meaningful risk variables that we want to account for…” (Ross et al., 2023). Only Denver’s GRID and LA’s GRYD captured information about gang member anti-social behaviors, while the others only documented identity or status.

### Gun violence exposure/victimization

Of the seventeen evaluations that documented participants’ prior gun violence exposure, eleven asked whether the participant had used a gun, suffered a gunshot injury or had witnessed a gun homicide. Only READI, GRID, GRYD, Oakland’s Unite, Philadelphia’s GVI, and Trinidad and Tobago’s Cure Violence explicitly documented whether participants were/are using guns. For example, the GRID program asked participants whether they had used a gun to shoot someone and whether someone had used a gun to shoot at them in the past three months. Cure Violence in Trinidad and Tobago asked participants whether someone close to them was a recent victim of a shooting and Safe Streets in Baltimore documented whether participants’ siblings had been shot or shot at. The D.C. Cure Violence evaluation asked whether participants had access to a weapon, but not necessarily if they were using it to resolve conflicts or conduct criminal activity.

### Social & environmental risks

Data on participants’ experiences with being a victim again varied widely among programs and were not limited to gun violence victimization. Baltimore’s Safe Streets asked about prior family victimization and if a participant had trouble seeking drug or alcohol rehab, finding a job, or managing emotions, flashbacks, anxiety and/or nightmares. LA GRYD noted if the participant had poor parental supervision, exhibited early childhood aggression, antisocial behaviors, if the client lived in an unstable home (i.e., foster/group care), used drugs or alcohol, or had documented mental health issues. GRYD also uses its Youth Services Eligibility Tool (YSET) to determine participant eligibility, and clients must meet or exceed risk in at least four of the following domains: Weak Parental Supervision, Critical Life Events, Impulsive Risk-Taking, Guilt Neutralization, Negative Peer Influences, Peer Delinquency, Family Gang Influence, and Self-Reported Delinquency. Boston’s ROCA documented whether participants were using medication to control a mental health issue. Chicago’s READI used their own risk assessment tool that asked: “whether the person was a victim of a violent crime; had previously been incarcerated for a gun-related offense; was an active member of a street gang; had substance abuse issues; was unstably housed; was promoting violence on social media; and had recently been arrested. Outreach workers were instructed that a referral had to meet, at a minimum, more than one of these conditions (Bhatt et al., 2024, p.41).” Denver’s GRID summarized multiple types of individual-level victimization experiences into a ‘victimization variety score.’

### Receiving supportive services

We found that only five of the evaluations we reviewed included information about whether clients were receiving any services prior to enrollment. Boston’s ROCA asked whether participants had a history of receiving any counseling and if they were currently receiving public assistance such as housing, healthcare, food, social welfare or any other public assistance. Denver’s GRID program documented whether participants were enrolled in a gang reduction program, mental health, addiction, education, employment, or extracurricular activity, or engaged by any public, private, or non-profit human service organizations. LA’s GRYD program documented whether participants were previous enrolled in substance abuse or mental health treatment. Oakland’s Unite documented if participants had ever received life coaching, self-sufficiency supports, and/or violent incident crisis response.

### Assets/strengths

Only two evaluations reported on program participants’ assets or strengths. Denver’s GRID noted if participants were interested in job training. LA’s GRYD documented if participants regularly attended meetings, were engaged in any prosocial activities, demonstrated any independence from social or gang influences, if they came from a family that enforced rules and roles, if they were close with family and if they had a knowledge of and pride in their family’s history. The GRYD program was also the only program we found that asked whether a participant had traveled outside a three-mile radius from their home for prosocial activities in the prior six months.

## Discussion

Our findings suggest that few CVI programs include detailed information about the range of known risk factors for gun violence facing their participants in published evaluations. This lack of data can make it difficult to ascertain how exactly these programs determine what it means for an individual to be ‘at high risk for gun violence.’ We found that very few CVI programs are measuring known individual or neighborhood-level risk factors for gun use and that even fewer are asking participants about the presence of potential protective factors (the exceptions being Denver GRID and LA GRYD). Available risk assessment tools and validated metrics, such as the SaFETy score, VRRAI and ACEs, were not widely adopted.

Our findings suggest that that many known risk factors for gun violence were hardly mentioned in the CVI evaluations we reviewed. Only six of the CVI evaluations offered specific information about participants’ level of acute or chronic poverty, whether and where they were employed and if they had a steady income. Yet, the field of firearm violence prevention has revealed that increasing one’s economic stability and security and reducing financial stress are significantly associated with reductions in firearm violence (Rowhani-Rahbar et al., 2022). Only LA GRYD and Boston ROCA asked about participants’ housing insecurity, prior homelessness, living in a shelter or related experiences. Yet, homelessness, chronic housing insecurity and experiencing shelter insecurity can contribute to firearm violence (Loe et al., 2025; Stansfield & Semenza, 2023). Similarly, youth who experience chronic food insecurity, a risk factor not measured by any CVI evaluation we reviewed, may be more likely to engage in gun violence (Ali et al., 2022; Ghio et al., 2025; Smith et al., 2020). The role of social media in instigating and perpetuating gun violence was only mentioned by one CVI evaluation (READI) we reviewed. However, recent scholarship has recognized social media as a key risk factor, shedding light on the role that these platforms have come to play as the digital extension of spaces where violence is expressed, processed, and escalated (Garcia Whitlock et al., 2023; Sichel et al., (Sichel 2021); Patton et al., 2018).

When CVI programs do measure client risk, measurement and reporting vary widely, as some include detailed descriptive statistics while others use general terms such as ‘most’ clients. We did find that some CVI programs are gathering detailed information about participants’ prior risky exposures, such as LA GRYD, Denver’s GRID, Baltimore’s Safe Streets, Boston’s ROCA, and numerous programs in Chicago. Yet, even among these programs, client risk is measured differently. We were surprised that more evaluations did not measure whether or not their clients were currently using guns or make clear which risk factors they suspected might contribute to their participants’ likely use of guns in the future. Prior and current gun use is one of the strongest predictors of future gun violence (Huesmann, et al., 2021). Further, we found no evidence that CVI evaluations reported whether enrolled participants were from conflicting/rival groups/gangs in their respective cities. We only learned if a client was group or gang affiliated. This suggests that some CVI programs might be only engaging clients from one side of the ‘warring’ parties in a city.

While some CVI programs evaluated the correlation between their intervention and reductions in gun-use risk factors among participants, these same programs mostly excluded any mention of protective or buffering factors. We found that very few CVI programs seem to be measuring the presence or absence of known protective factors for their clients, such as the number of healthy adults in their lives, access to mental and physical health care, employment and artistic skills or knowledge, and positive peer or other relationships.

Our findings suggest that in the absence of a detailed and nuanced understanding of participants’ risk and protective factors, CVI evaluations may not be able to document which interventions are effective in changing participant’s behaviors. These same evaluations may report a change in gun violence in a place or among a specific group, but few are reporting if the risk and or protective factors – which we have argued can help us understand if a person may or may not use a gun to resolve a conflict – of the people they engaged had or had not changed. Without this information, we are left unsure if the interventions themselves helped change participants and if so, in what ways?

These findings also suggest that street outreach-based CVI could benefit from a more standardized approach to documenting and reporting participants’ risk and protective factors. A greater understanding of participants’ baseline characteristics could serve two critical purposes. First, it has the potential to help the emerging field of CVI gain a more comprehensive understanding of participant’s needs at baseline, and the assets in their lives that can be leveraged to promote behavioral change. Second, it would strengthen the rigor of the evaluations by helping identify the causal mechanisms responsible for the observed reductions in violence at the community level. Without a more systematic understanding of individual-level characteristics and changes, the field faces challenges in assessing which services are effective for whom, which risks are successfully mitigated, and the mechanisms by which CVI disrupts the cycles of violence.

As the field of CVI develops, we invite practitioners and researchers in this space to collaboratively reach consensus on a core set of risk and protective measures to document in CVI evaluations. We contend that doing so would enable evaluations to more comprehensively assess whether and how unique interventions and services may have contributed to violence prevention, while being considerate of the distinctive features of each CVI program. This paper lays the groundwork for this conversation by documenting current practices in reporting participant risks and protective factors at baseline. Drawing on our analysis of how risk and protective factors for gun use are characterized in evaluations, we propose an initial set of key domains for participant data collection and reporting that street outreach-based CVI programs should consider:

- Descriptive: Age, demographics, gender, neighborhood location and existing level of violence, individual and family poverty level;
- Risks: Adverse Childhood Experiences, prior gun use and exposure to firearms (i.e., peers, family, witnessed gun homicide, etc.), group affiliation;
- Assets: Openness to change, educational and employment histories, interpersonal skills, other strengths and assets.

### Limitations

There are limitations to this review in addition to those we made explicit in the methods section. First, we acknowledge that many of the evaluations included in this review were designed to evaluate community-level outcomes such as neighborhood and city-level violence rates or changes in group norms/behaviors – not individual-level outcomes. Second, we only reviewed publicly reported evaluations. We acknowledge that some programs may be collecting more comprehensive individual-level data but are choosing to limit its publication due to confidentiality and data privacy concerns. These are important considerations given that CVI participants may be frequently targeted by the criminal legal system. Finally, we acknowledge that the absence of individual-level data may reflect the well-documented challenges of building trusting relationships with participants who may be skeptical of intervention programs and therefore reluctant to provide detailed information about their personal histories, lived experiences, and backgrounds (Bocanegra & Aguilar,, 2024; Butts et al., 2015; Hureau & Papachristos, 2024; Ross et al., 2023).

## Conclusion

More work is needed to better understand how CVI programs are measuring risk among program participants. The literature suggests that the risks behind gun use among urban youth are heterogeneous and may vary by a host of individual and community-level factors. CVI programs appear to be largely measuring participants’ risk based on demographic and prior criminal histories, while missing the roles of trauma, ACEs, and other social and contextual factors. When measured, these factors might shift CVI to better gauge the ‘readiness’ of clients for specific interventions, not just their risk of perpetuating violence. Understanding risks and protective factors might also contribute to more evaluations that detail which interventions support which client behaviors that might help stem the epidemic of urban gun violence.

## Data Availability

No datasets were generated or analysed during the current study.

## **Appendix A**

### Complete search strategy

(("Community Violence Intervention" OR "Group Violence Intervention" OR"violence interrupt*" OR "credible messenger*" OR CVI OR (("ceasefire" OR "operation ceasefire") AND ("violence" OR "community" OR "intervention" OR"program")) OR "cure violence" OR "focused deterrence" OR "conflict mediation" OR "violence prevention outreach" OR "community-based violence prevention")

AND (eval* OR "program assessment" OR "intervention assessment" OR "outcome evaluation" OR "impact evaluation" OR "process evaluation" OR "program evaluation" OR "program effectiveness" OR "intervention effectiveness" OR "program efficacy" OR "intervention efficacy" OR "program outcomes" OR "intervention outcomes" OR "program impact" OR "intervention impact")

NOT ("hospital-based" OR "hospital based" OR HVIP OR"emergency department" OR "emergency room" OR "trauma center" OR "medical center" OR "clinical setting" OR"mental health" OR "War" OR "HIV" OR"domestic violence" OR "intimate partner violence"))
